# Intraperitoneal administration of the anti-IL-23 antibody prevents the establishment of intestinal nematodes in mice

**DOI:** 10.1038/s41598-018-26194-x

**Published:** 2018-05-17

**Authors:** M. Gomez-Samblas, D. Bernal, A. Bolado-Ortiz, S. Vilchez, F. Bolás-Fernández, A. M. Espino, M. Trelis, A. Osuna

**Affiliations:** 1 Instituto de Biotecnología, Grupo de Bioquímica y Parasitología Molecular, Departamento de Parasitología, Universidad de Granada, Campus Universitario Fuentenueva, 18071 Granada, Spain; 2 Departament de Bioquímica i Biologia Molecular, Universitat de València, C/Dr. Moliner, 50, 46100 Burjassot, Valencia Spain; 3 Departament de Farmàcia i Tecnologia Farmacèutica i Parasitologia, Àrea de Parasitologia, Universitat de València, Av. V.A. Estellés, s/n, 46100 Burjassot, Valencia Spain; 4 Joint Research Unit on Endocrinology, Nutrition and Clinical Dietetics, Health Research Institute-La Fe, Universitat de Valencia, Av. Fdo. Abril Martorell, 106, 46026 Valencia, Spain; 5 Instituto de Biotecnología, Grupo de Bioquímica y Parasitología Molecular, Departamento de Bioquímica, Universidad de Granada, Campus Universitario Fuentenueva, 18071 Granada, Spain; 6 Departamento de Microbiología y Parasitología, Facultad de Farmacia, Universidad Complutense, Plaza de Ramón y Cajal s/n. Ciudad Universitaria, 28040 Madrid, Spain; 7 Laboratory of Immunology and Molecular Parasitology, Department of Microbiology, University of Puerto Rico, School of Medicine, PO BOX 365067, San Juan, 00936-5067 Puerto Rico

## Abstract

Previous studies have established that an increased Th-9 response creates a hostile environment for nematode parasites. Given that IL-23, a cytokine required for maintenance of the IL-17–secreting phenotype, has inhibitory effects on IL-9 production, we hypothesized that reducing circulating IL-23 by treatment with anti-IL-23 antibodies would reduce the establishment and development of parasitic intestinal nematodes. In this study, we show that animals treated with anti-IL-23 monoclonal antibodies showed a drastic reduction in the number of mouse pinworms (*Aspiculuris tetraptera)* recovered from the intestine (p < 0.001) at 23 days post-infection compared to the untreated animals. The cytokine levels in Peyer’s patches (PP) in treated and infected animals increase the expression of interleukins such as IL-25, IL-21, and IL-9, augmenting mucus production in the crypts, and boosting chemokines, such as OX40 and CCL20 in the mucosa. Our results suggest that the Th17/Th2 regulatory mechanism provoked by the administration of the anti-IL-23 antibody prevents the implantation of the intestinal nematode in mice. The diminished inflammatory IL-17 levels alter the Th9 environment perhaps as a consequence of IL-17 inhibiting IL-9 expression. These Th9 conditions may explain the successful treatment against Inflammatory Bowel Disease (IBD) both with antibodies against IL-23 or through parasitization with nematodes.

## Introduction

Parasitism by intestinal nematodes triggers a strong Th2 response in the host, with an increase in interleukin markers of this response, such as IL-4, IL-5, IL-10, IL-15^1,2^. The stimulation of Th2 by helminth infection diminishes the inflammatory intestinal processes^3^, and therefore helminth infections have been used to treat inflammatory intestinal diseases as an alternative to treatment with antibodies against IL-23^4–8^. This Th2 stimulation has recently been confirmed to be the result of the induction of IL-25 by tuft cells^9,10^. This interleukin, also called IL-17E, induces IL-13, which stimulates the production of interleukins involved in the Th2 response^11,12^. IL-25 production also precedes the rise in IL-9 levels in the parasite-expulsion process, with IL-9 being ultimately responsible for eliminating the nematodes^13,14^ by inducing variations in the intestinal mucosal niche, such as increased mucus by goblet-cell activation^15^, greater intestinal contractibility^16^, and altered intestinal permeability^17^. In addition, IL-9 induces mast-cell expansion and secretion of specific proteases, such as mouse mast-cell protease 1 (mMCPT-1)^18,19^, as well as higher levels of CCL11 or eotaxin, the chemokine that specifically attracts eosinophils^20^.

Elliott *et al*.^21^, previously described that the levels of IL-17 are suppressed during nematode parasitism. Among other functions, IL-23 is involved mainly in promoting Th17 differentiation and proliferation^22^, helping to sustain IL-17 production by CD4^+^ T cells^23^ as well as repressing IL-9 production^24^, which as mentioned above is largely responsible for nematode expulsion by creating a hostile environment in the intestine. Thus, it is expected that decreasing the amount of circulating IL-23 should boost IL-25, IL-13 and IL-9 production and consequently modifies the physiological conditions suitable for the establishment of gastrointestinal nematodes through the stimulation of the hostile conditions described above.

For this study, we have employed the mouse pinworm *Aspiculuris tetraptera*, a nematode with a direct life cycle that induces little damage to the host epithelium and does not trigger a host inflammatory response^25^. Using this model, we injected an anti-IL23 monoclonal antibody in mice prior to a challenge infection with embryonated eggs of *A. tetraptera* to determine whether the reduction of circulating IL-23 could prevent the successful implantation of parasite in the intestine. The parasitological data both of nematode establishment as well as development rates have been studied, comparing the results to control (untreated mice). Similarly, the different interleukin expression levels in spleen, mesenteric lymph nodes (MSN), and Peyer’s patches (PP) were analyzed, together with other parameters, including chemokines in the intestinal mucosa in the different mice groups (treated with the anti-IL-23 antibodies and infected, treated but uninfected and control infected mice).

The data compiled corroborate that Th17 and specifically IL-17 are needed to maintain the immunological and physiological environment conducive to nematode establishment in the intestine. The IL-23, has inhibitory effects on IL-9 production^21,25^. The decrease in the normal levels of this interleukin provide a Th9 environment that impedes the nematode establishment in the intestine and may account for the treatment against inflammatory bowel diseases (IBD) both with antibodies against IL-23 as well as by nematode parasitism. This suggests that a higher level of intestinal IL-9 would permit treatment against these chronic inflammatory bowel diseases.

## Results

In order to investigate whether decreasing circulating IL-23 levels could prevent establishment and development of nematode parasites, we determined the *A. tetraptera* recovery rates from the intestine of the infected control mice (C-IF) and those inoculated and infected with the IL-23^mAB^ (AB-IF). In the infected control (C-IF), values reached 11.4 ± 9.9, while for infected mice treated with anti-IL-23 (AB-IF), in which IL-23 was depleted before and during the first days of infection, rates were 0.55 ± 0.45 with a parasitism reduction of 98.2%.

**Figure 1:**
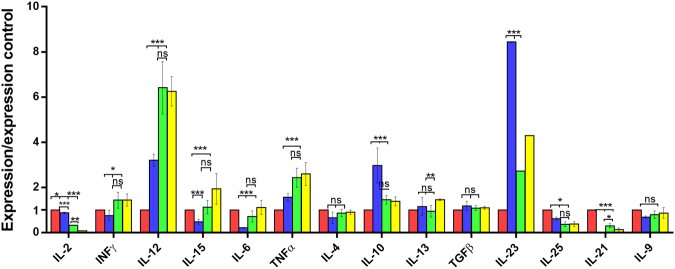
Expression levels of cytokines in spleen determined by quantitative real time PCR after normalization with β-actin, and standardization against control animals. Red bar, C (uninfected and untreated control group); Blue bar, C-IF (infected control group); Green bar, AB-IF (animal treated with IL-23^mAB^ and infected with *A. tetraptera* group); Yellow bar, AB-C (treated with IL-23^mAB^ and uninfected group). The values are the means of the normalized expression values ± SEM. Tukey Test, p < 0.001 (***) and p < 0.05 (*).

**Figure 2:**
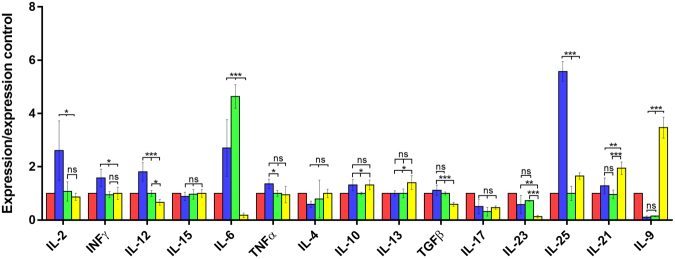
Expression levels of cytokines in MSN determined by quantitative real time PCR after normalization with β-actin, and standardization against control animals. Red bar, C (uninfected and untreated control group); Blue bar, C-IF (infected control group); Green bar, AB-IF (animal treated with IL-23^mAB^ and infected with *A. tetraptera* group); Yellow bar, AB-C (treated with IL-23^mAB^ and uninfected group). The values are the means of the normalized expression values ± SEM. Tukey Test, p < 0.001 (***) and p < 0.05 (*).

**Figure 3:**
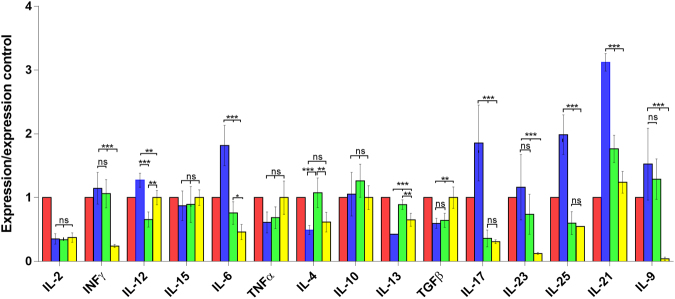
Expression levels of cytokines in PP determined by quantitative real time PCR after normalization with β-actin, and standardization against control animals. Red bar, C (uninfected and untreated control group); Blue bar, C-IF (infected control group); Green bar, AB-IF (treated with IL-23^mAB^ and infected group); Yellow bar, AB-C (treated with IL-23^mAB^ and uninfected group). The values are the means of the normalized expression values ± SEM. Tukey Test, p < 0.001 (***) and p < 0.05 (*).

The results of the cytokine expression analysed by qPCR in the different groups of mice for lymphoid tissues: spleen, MSN, and PP are presented in Figs 1, 2, 3, showing that the IL-23 levels in the IL-23^mAB^ treated groups were significantly lower than in the infected control group (C-IF) in the three lymphoid tissues studied.

The relative expression of cytokines in the spleen (Fig. 1) indicates that the experimental infection (C-IF) increases the expression of IL-10 and IL-23, being significantly higher than in the other groups, while the expression of IL-15 (***), INF-γ (*), IL-6 (***), and TNF-α (***) and IL-12 (***), the last three corresponding to a Th1 response, were significantly lower than in the other groups.

When the IL-17 expression levels in PP were analysed by qPCR (Fig. 3), C-IF mice showed a significant increase [p < 0.001 (***)] with respect to the other groups, while in the other groups, the expression levels were lower and no significant differences were observed when compared to each other.

**Figure 4:**
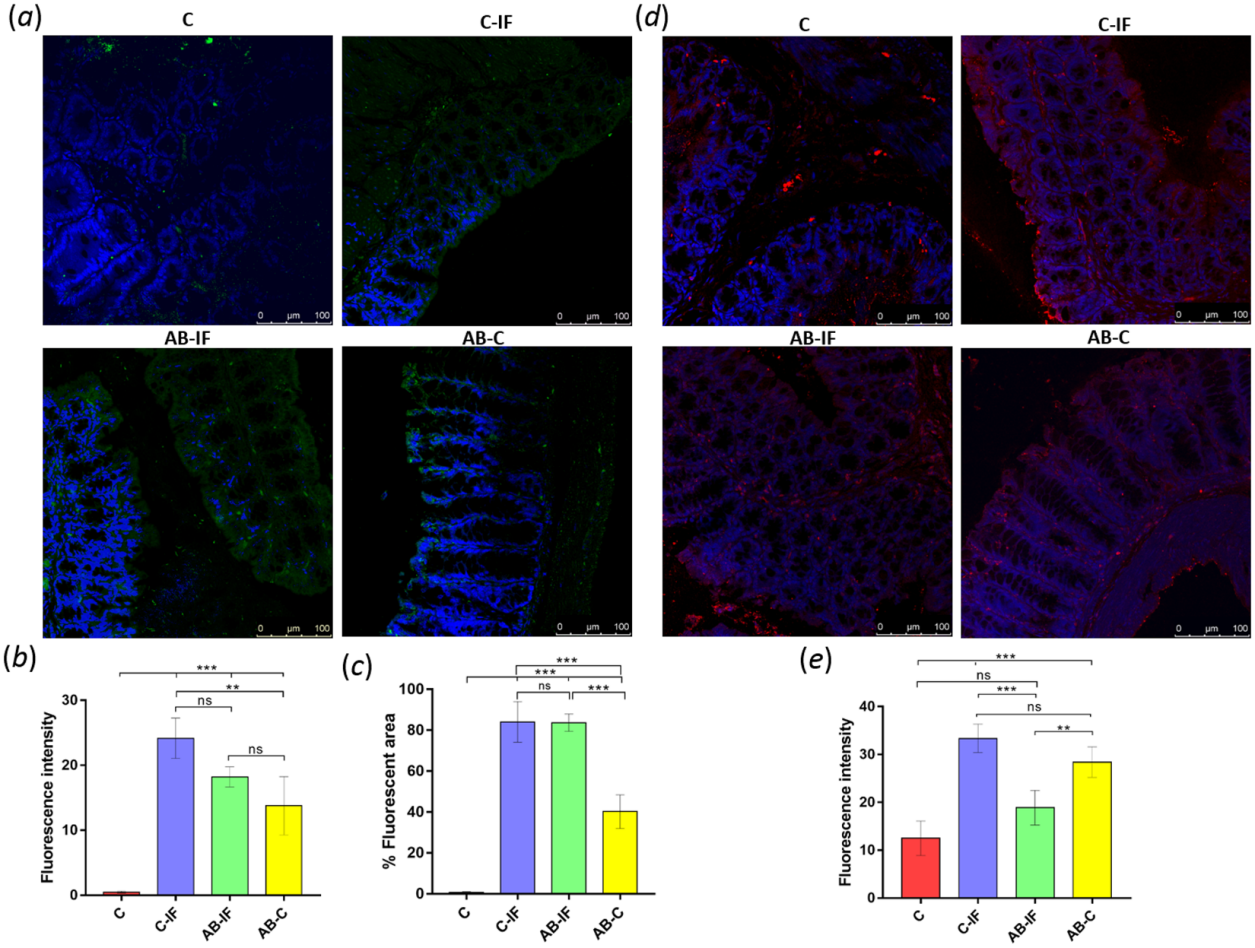
Immunofluorescence location and quantification of IL-17 and CCL2 by confocal microscopy in colon sections of mice used in the experiments. C (uninfected and untreated control group); C-IF (infected control group); AB-IF (treated with IL-23^mAB^ and infected group); AB-C (treated with IL-23^mAB^ and uninfected group). (**a**) Semi-thin intestinal sections showing green (FITC) signal for anti-IL-17 labelled antibody. The cell nuclei were stained with DAPI (blue). (**b**) Measurement of fluorescence intensity for IL-17 in four different areas of 100 µm^2^, mean values ± SEM. (**c**) % Fluorescent area for IL-17 in four different areas of 100 µm^2^, mean values ± SEM. (**d**) Semi-thin intestinal sections showing red (Alexa Fluor^®^ 594) signal for the labelled anti-CCL2 antibody. The cell nuclei were stained with DAPI (blue). (**e**) Measurement of fluorescence intensity for CCL2 in four different areas of 100 µm^2^, mean values ± SEM. The values are the means of the normalized expression values ± SEM. Tukey Test, p < 0.001 (***) and p < 0.05 (*).

The IL-17 levels in the intestinal mucosa determined by confocal laser microscopy reflect that the maximum fluorescence intensity for this interleukin appears attached to the border of the intestinal mucosa in all infected (C-IF and AB-IF) and IL-23^mAB^ uninfected (AB-C) groups, with no fluorescence observed in the group of uninfected control animals (C) (Fig. 4a,b). The percentages of the fluorescent area indicate (Fig. 4c) that the groups of infected animals (C-IF) have similar fluorescent areas with approximately 82% fluorescence in the area of the section analyzed, similar to the AB-IF group, whereas only 40% of the surface area was fluorescent in the AB-C group.

Low IL-21 expression levels appeared in the spleen of the C-IF group (***), and those treated with anti-IL-23 (AB-IF and AB-C) in comparison with the expression shown in the other lymphoid tissues studied (Fig. 1). However, high levels appeared in the PP in the C-IF group and to a lesser extent in the AB-IF group (Fig. 3).

The IL-15 and IL-6 expression appeared high (***) in the spleen in the AB-C group whereas, in the MSN, IL-6 registered the highest expression (***) in the AB-IF group (Figs 1 and 2). Meanwhile, the PP in the C-IF group registered the highest level of expression for IL-6 vs. the groups treated with the IL-23^mAB^ (AB-IF and AB-C) (Fig. 3).

**Figure 5:**
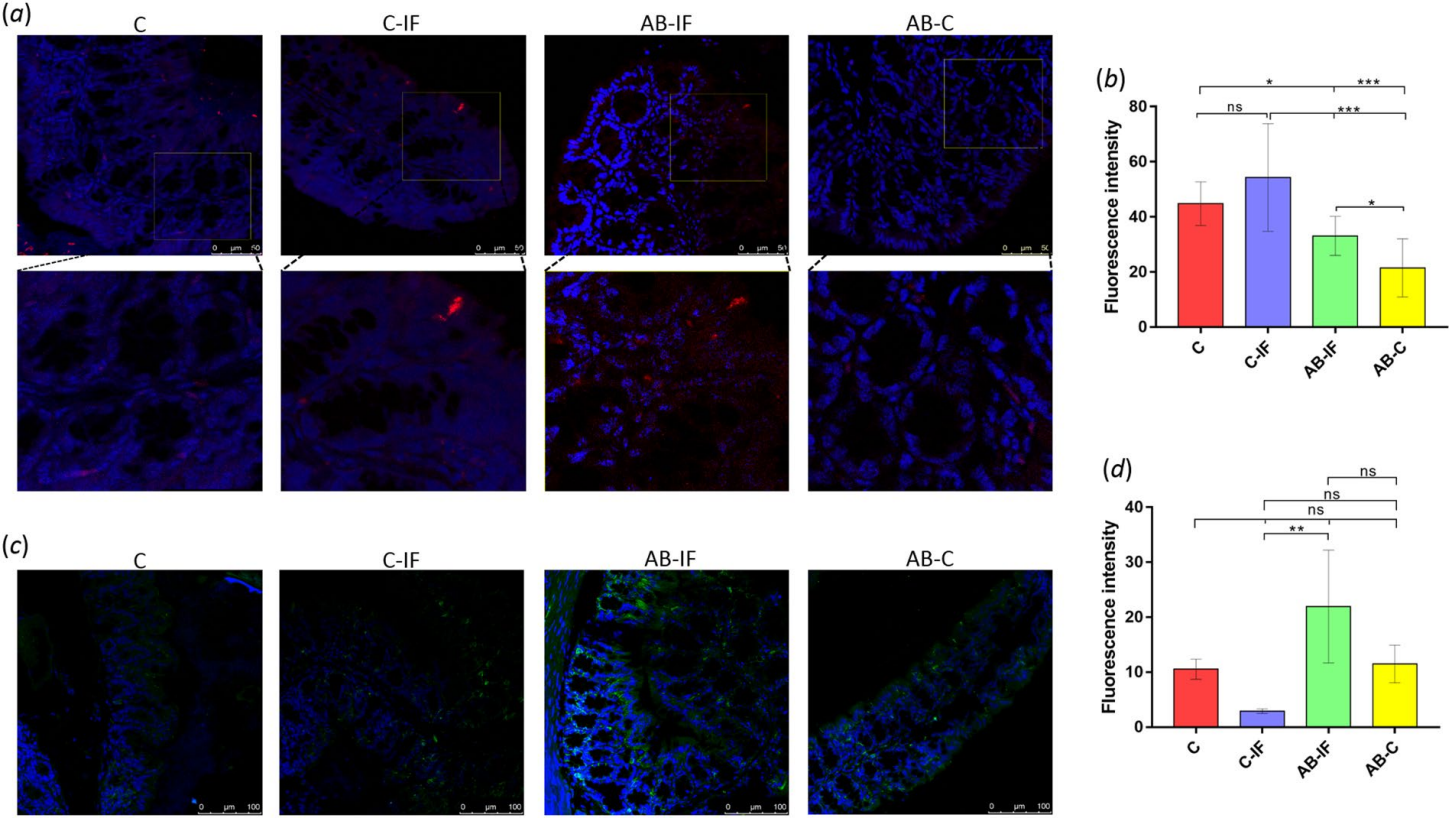
Immunofluorescence location and quantification of tuft cells (anti-DCAMKL-1) and plasma cells (anti-CD138) by confocal microscopy in the colonic sections of mice used in the experiments. C (uninfected and untreated control group); C-IF (infected control group); AB-IF (treated with IL-23^mAB^ and infected group); AB-C (treated with IL-23^mAB^ and uninfected group). (**a**) Semi-thin intestinal sections showing red (Alexa Fluor^®^ 647) cells labelled with anti-DCAMKL-1 antibody. The cell nuclei were labelled with DAPI (blue). (**b**) Measure of fluorescence intensity of all tuft fluorescent cells, mean ± SEM. (**c**) Semi-thin intestinal sections showing green (FITC) cells labelled with anti-CD138 antibody. The cell nuclei were labelled with DAPI (blue). (**d**) Measure of fluorescence intensity of plasma cells in four areas of 100 µm^2^, mean ± SEM. The values are the means of the normalized expression values ± SEM. Tukey Test, p < 0.001 (***) and p < 0.05 (*).

The IL-25 expression was significantly higher (***) in the C-IF group in the PP and MSN when compared to the other experimental groups, with the minimum values of this interleukin in the spleen measurements (Figs 1–3). These results agree with data on the intensity of the fluorescence of the tuft cells, as shown in Fig. 5a,b.

The IL-13 expression was lower in the C-IF with respect to treated groups in the PP, but the AB-C group showed high expression values with respect to the other groups in the spleen (**) and in the MSN (*) (Figs 1–3).

Regarding the results for IL-9 expression levels, we did not observe significant differences in the spleen (Fig. 1). However, in the MSN, we observed a marked increase (***) in the uninfected animals treated with anti-IL-23 (AB-C) (Fig. 2). In contrast, when we investigated the PP, the C-IF and AB-IF groups showed the highest expression levels, with respect to the uninfected C and AB-C groups (Fig. 3).

Immunohistochemistry analyses of cytokine CCL2 for the different groups are shown in Fig. 4d and e. The highest fluorescence levels were found in the infected control group (C-IF), followed by the groups where IL-23 was neutralized (AB-C and AB-IF), and the uninfected control (C). No significant differences (ns) were observed between the infected groups (C-IF and AB-IF), while the differences between the C, C-IF and AB-C groups, and between the two groups in which IL-23 was neutralized were statistically significant (p < 0.001 (***) and p < 0.05 (**), respectively).

**Figure 6:**
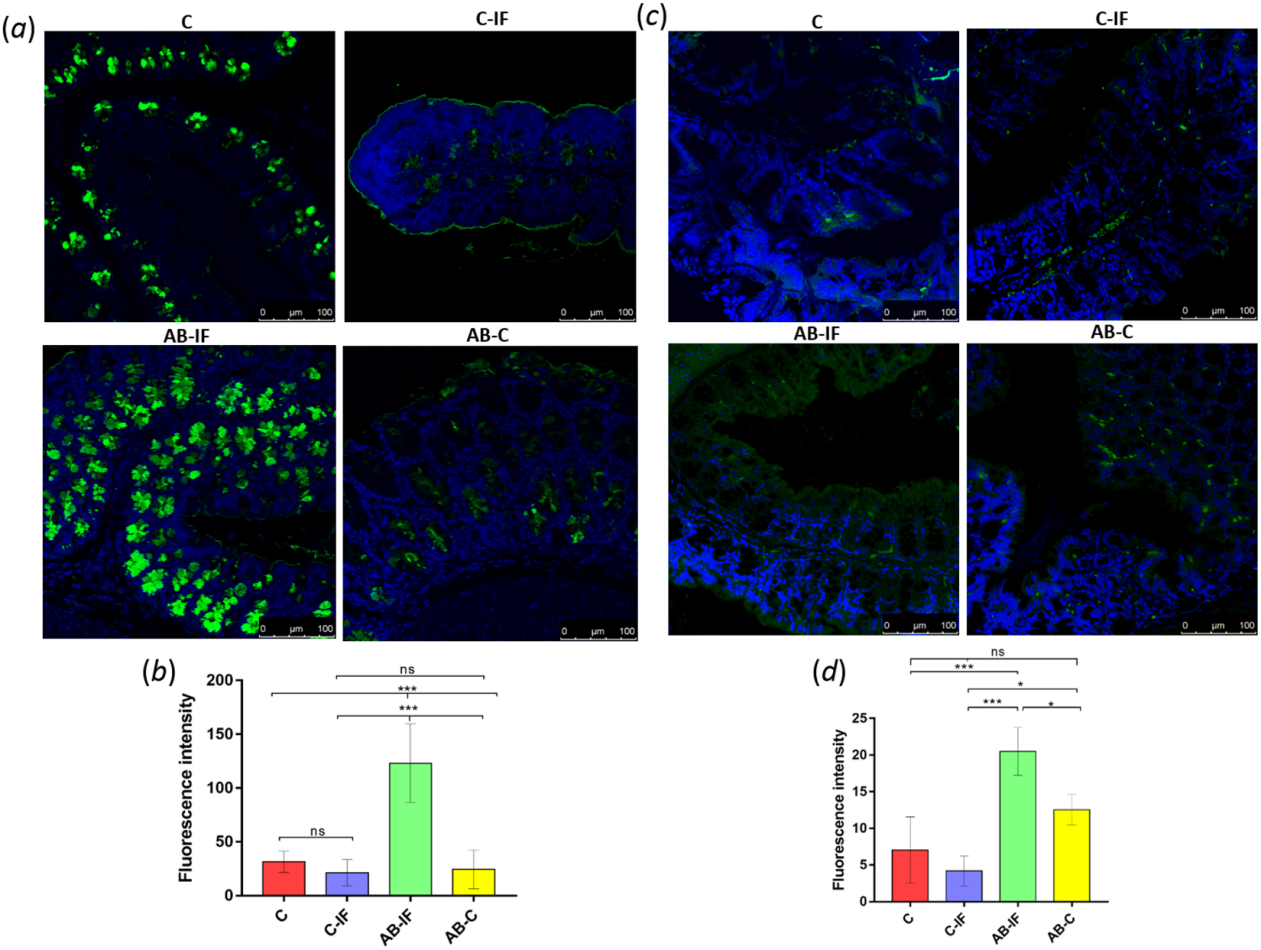
Immunofluorescence location and quantification of mucus and OX40 by confocal microscopy in the colon sections of mice used in the experiments. C (uninfected and untreated control group); C-IF (infected control group); AB-IF (treated with IL-23^mAB^ and infected group); AB-C (treated with IL-23^mAB^ and uninfected group). (**a**) Semi-thin intestinal sections showing mucus in the crypts using WGA lectin labelled with FITC (green). The cell nuclei were stained with DAPI (blue). (**b**) Measure of fluorescence intensity for mucus in all fluorescent crypts, mean ± SEM. (**c**) Semi-thin intestinal sections showing FITC green signal for the labelled anti-OX40 antibody. The cell nuclei were stained with DAPI (blue). (**d**) Measure of fluorescence intensity for OX40 in four different areas of 100 µm^2^, mean ± SEM. The values are the means of the normalized expression values ± SEM. Tukey Test, p < 0.001 (***) and p < 0.05 (*).

The results of the confocal laser microscopy study investigating the production of mucus in the intestinal crypts of the mice of the different groups are shown in Fig. 6a. The levels of fluorescence intensity of labelled WGA (Fig. 6b) were significantly higher (p < 0.001 (***)) in the AB-IF group with respect to the C, C-IF and AB-C groups, while no significant differences were observed among the other groups studied.

**Figure 7:**
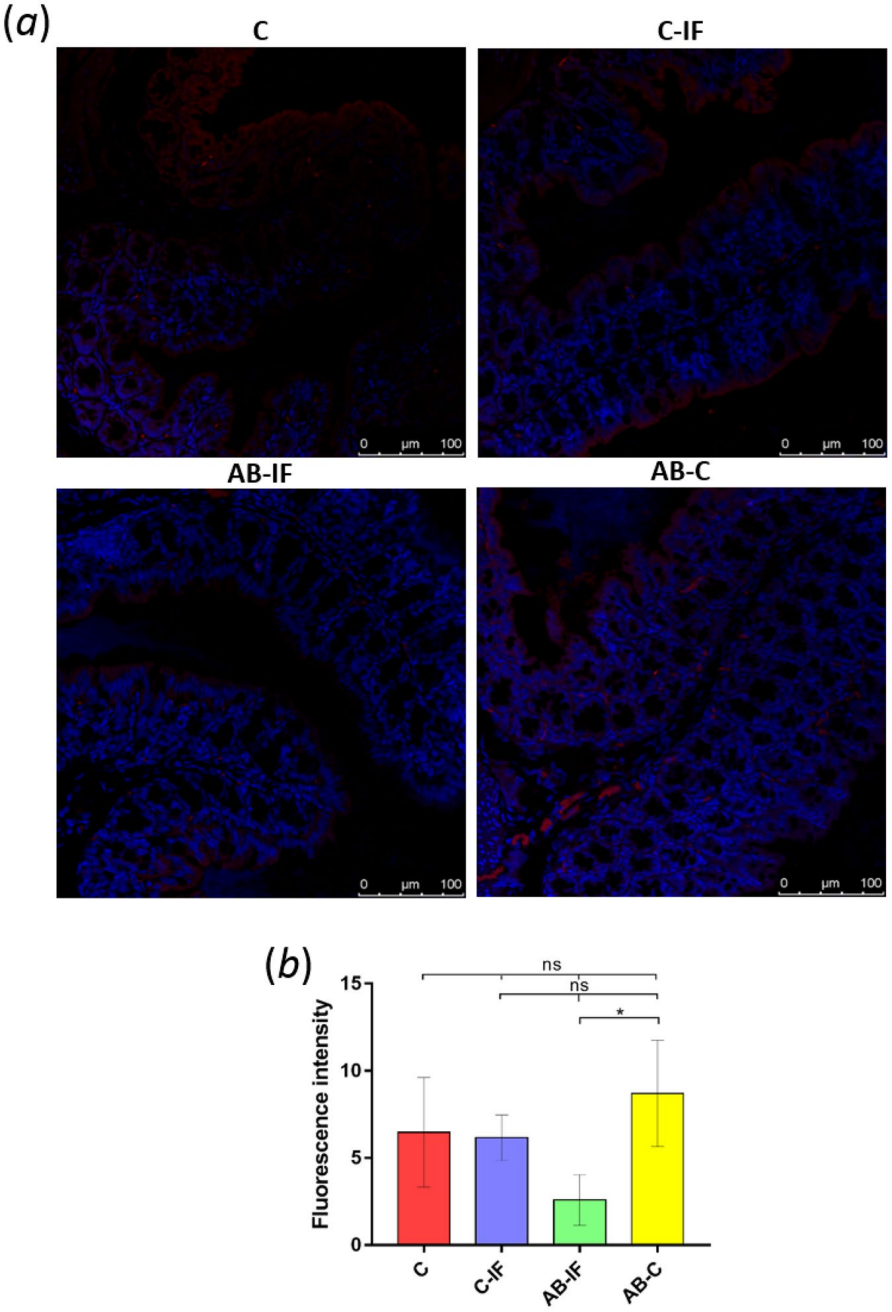
Immunofluorescence location and quantification of CCL17 by confocal microscopy in the colon sections of mice used in the experiments. C (uninfected and untreated control group); C-IF (infected control group); AB-IF (treated with IL-23^mAB^ and infected group); AB-C (treated with IL-23^mAB^ and uninfected group). (**a**) Semi-thin intestinal sections showing red (Alexa Fluor^®^ 594) signal for the labelled anti-CCL17 antibody. The cell nuclei were stained with DAPI (blue). (**b**) Measure of fluorescence intensity for CCL17 in four areas of 100 µm^2^, mean ± SEM. The values are the means of the normalized expression values ± SEM. Tukey Test, p < 0.001 (***) and p < 0.05 (*).

The results of the immunohistochemistry analysis of colon tissue using an antibody against the cytokine CCL17 are shown in Fig. 7a. The CCL17 signal is observed around the intestinal villi. The highest fluorescence levels (Fig. 7b) appeared in the AB-C group, with significant differences (p < 0.01 (*)) when compared to the AB-IF group. This latter group registered the lowest value for fluorescence intensity, although it was not significant with respect to the C and C-IF groups.

The results from similar immunohistochemical studies analysing the levels of OX40 are shown in Fig. 6c,d, indicating an increase in the fluorescence levels of this cytokine in the groups where IL-23 was neutralized (AB-IF and AB-C).

**Figure 8:**
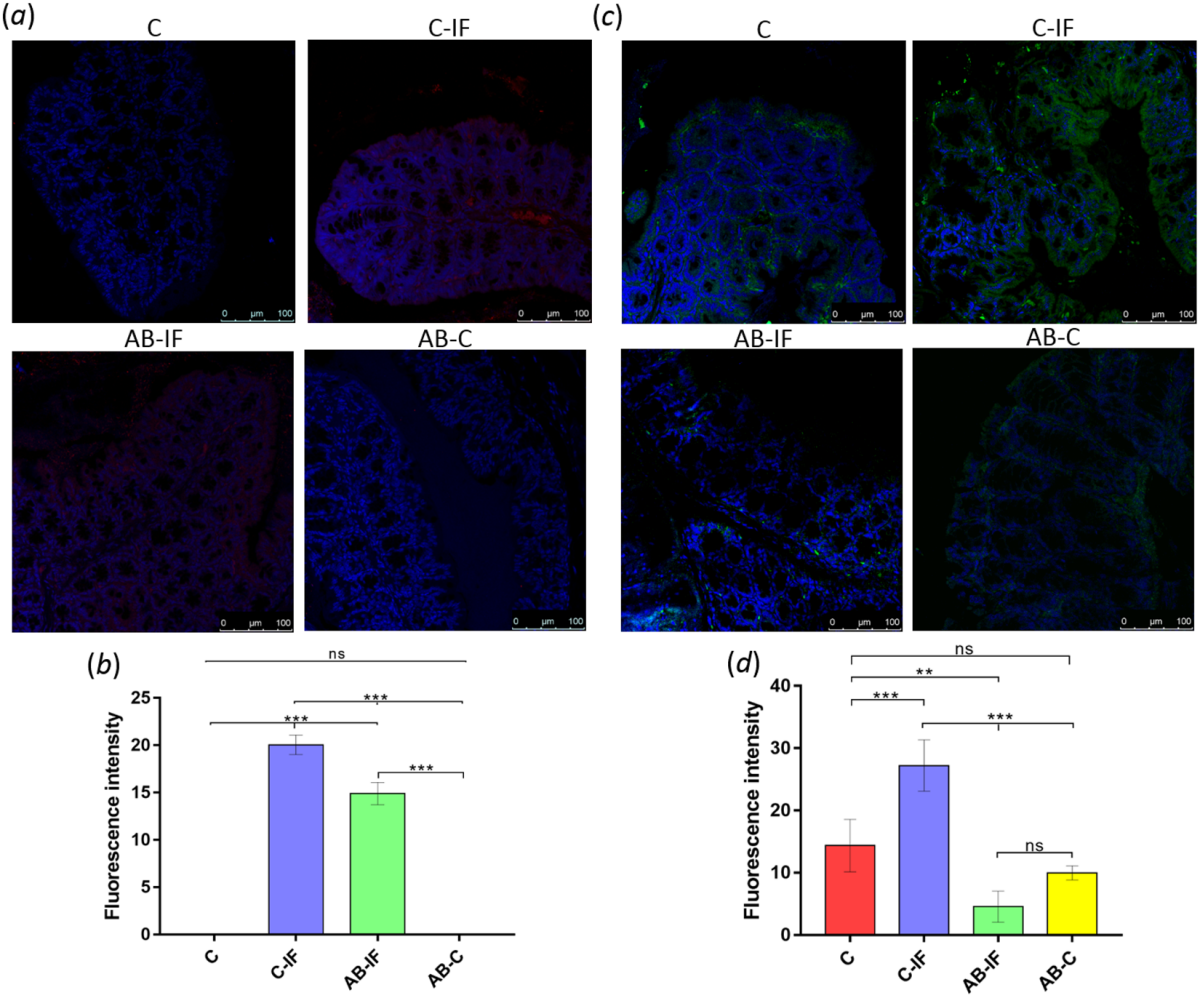
Immunofluorescence location and quantification of CCL20 and GRO by confocal microscopy in the colonic sections of mice used in the experiments. C (uninfected and untreated control group); C-IF (infected control group); AB-IF (treated with IL-23^mAB^ and infected group); AB-C (treated with IL-23^mAB^ and uninfected group). (**a**) Semi-thin intestinal sections showing red (Alexa Fluor^®^ 594) signal for the labelled anti-CCL20 antibody. The cell nuclei were stained with DAPI (blue). (**b**) Measure of fluorescence intensity for CCL20 in four areas of 100 µm^2^, mean ± SEM. (**c**) Semi-thin intestinal sections showing green (FITC) signal for anti-GRO antibody. The cell nuclei were labelled with DAPI (blue). (**d**) Measure of fluorescence intensity for GRO in four areas of 100 µm^2^, mean ± SEM. The values are the means of the normalized expression values ± SEM. Tukey Test, p < 0.001 (***) and p < 0.05 (*).

The CCL20 levels were also analyzed in the different groups (Fig. 8a). The results indicate that the fluorescent signal (Fig. 8b) was appreciable only in the infected mice, which suggests that it is dependent on the presence of the parasite. Moreover, the C-IF group showed significantly higher expression than the mice treated with AB-IF.

Immunohistochemical analyses of GRO expression (Fig. 8c) indicate the presence of this chemokine on the borders of the villi and around the crypts. For each group of mice, the quantification of the fluorescence intensity measurements appears in Fig. 8d, where the maximum value was found in the C-IF group. In the AB-IF and AB-C groups, minimum levels were detected, with no significant differences (ns) between them.

We also determined the amount of plasma cells in these groups by employing an anti-CD138 antibody specific for this cell type. The results and quantification are shown in Fig. 8c and d, respectively. We observed the highest levels of plasma cells in the AB-IF group with significant differences (**) respect the C-IF group (Fig. 5d).

Similar analyses examining the amount of tuft cells appear in Fig. 5a. In this case, the DCAMKL-1 antibody was used. The confocal microscopy images are presented in Fig. 5a and the quantification of the results in 8b. As depicted in the figure, we observed the lowest accumulation of tuft cells in the groups treated with the IL-23 antibody, especially the AB-C group. The C-IF group presented the highest values of fluorescence significant (***) with respect to the IL-23^mAB^ groups.

## Discussion

The extreme reduction in the recovery rates of the nematode *A. tetraptera* in the colon of the infected mice treated with IL-23^mAB^ indicates the requirement of this interleukin for the normal establishment of the nematodes in the host intestine. The neutralization of this interleukin, necessary for the maintenance of the Th17 response, may lead to intestinal changes that alter the optimal conditions for the intestinal colonization and subsequent development of the nematode in the host colon. The very low number of intestinal worms detected in our experiments does not correspond to the normal decrease in the parasite load at 17–19 days post-infection, as assessed by Behnke in 1975^25^, which occurs naturally after massive infection. Therefore, our results indicate that the presence of IL-23 plays an essential role for the establishment of intestinal nematode parasites in their niche.

The intestinal mucosa, which is the first line of defence encountered by the nematodes, is a layer of mucus secreted by the goblet cells. It is made up primarily of mucins and different bioactive factors such as resistin, intelectin, and the calcium-activated chloride channel-3^15,26,27^ together with other molecules such as antibodies, defensins segregated by the Paneth cells and lysozymes that cover the entire intestinal epithelium^28^. All of these molecules are involved in the defence mechanisms against parasites and pathogenic bacteria^29,30^. The colonic mucosa, the site of *A. tetraptera* establishment, has two mucus layers: the inner layer that appears to be linked to the mucosa and the outer one, less dense, that is in contact with the parasites. Another possible defence mechanism of the mucus is the physical barrier itself, which interferes in nematode feeding and mobility. This would explain why nematode infections induce a hyperplasia of the goblet cells in the host^31^. According to our results, the group of infected mice pre-treated with the anti-IL-23 antibody had the highest (***) quantity of mucus in the crypts and reduced nematode load, whereas the rest of the groups, AB-C, C and C-IF, presented lower mucus production.

A recent study investigating the role played by the intracellular “Nod receptor” in goblet cell activation during the process of inflammation and its effect on the expulsion of *Trichuris muris*^31^ showed that the activation of mucus-producing goblet cells is mediated by the binomial response of the IL-25- IL-9 -IL-13^29,32–34^. IL-13 is the regulator of goblet cell hyperplasia. IL-13-overexpressing mice develop intestinal goblet cell hyperplasia and the administration of exogenous IL-25 or IL-9 induce goblet cell hyperplasia and increased mucin expression via IL-13 dependent pathways^35^.

IL-9 expression levels in the spleen showed no significant differences between the groups. The MSN registered a high level of IL-9 expression in the AB-C group. In the PP, the lymphoid organ most directly in contact with the parasite, the infected groups (C-IF and AB-IF) also presented the highest levels of IL-9 expression. This might indicate that IL-9 expression in the spleen is not a requirement for the alteration of the intestinal environment involved in the establishment of the nematodes in the gut, whereas increased expression of this interleukin in the MSN may be more important in this process.

IL-9 shows several pleiotropic effects, such us mucus production by the goblet cells, as mentioned above, and increased intestinal contractibility as a result of acetylcholine (Ach) production^36^. Ach increases the blood flow in the gastrointestinal tract, among other actions, boosting gastrointestinal muscle tone, intensifying the contractions of the cells of the smooth muscle that contribute to the expulsion of the worms, thus hindering parasite establishment. IL-9 induces alterations in intestinal permeability by reducing the expression of the Claudin 2 of the “tight junction” of the intestinal epithelium^37^ perhaps as consequence of the mast cell proteases (CD138)^38^. Transgenic mice overexpressing IL-9 display increased accumulation of mucosal mast cells and enhance intestinal permeability^18^, thus suggesting a functional role for this interleukin in the expulsion of the intestinal nematodes^36,39–43^. All these effects lead to the physical alteration of the niche, creating a hostile environment where the parasitic worms try to establish and are also involved in nematode resistance, thus explaining the hostile environment created upon induction of the Th9 response, after the treatment with the anti-IL-23 monoclonal antibody.

The expression of IL-9 is proposed to occur as a consequence of the stimulation of Treg and Th17 cells by IL-4 and TGF-β^44,45^. The negative regulatory effects between the IL-23 levels needed to maintain IL-17 production by the Th17 cells, and the production of IL-9 by the Th17 cells have been described elsewhere^24,46–48^. On the other hand, IFN-γ, a marker of the Th1 response, is known to be a negative regulator of the production of IL-9 as a consequence of the inhibitory effect of IFN-γ on IL-4 expression^49^. Moreover, IL-12 induces the production of IFN-γ and thus the levels of IL-12 and IL-9 should counterbalance each other^50^. IL-12 favours parasitism by nematodes, as has been reported in the adult phase of *Trichinella spiralis*, where recombinant IL-12 treatment of mice infected with the intestinal phase of the nematode *T. spiralis* reduces the expulsion of the adults from the intestine. These results indicate that a strong Th1 response, which would inhibit the Th9 response, favours parasite establishment^51^. In the PP, the expression of IL-12 in the AB-C group was higher than in the AB-IF group and this increase in the IL-12 levels were not accompanied by a higher IFN-γ expression, likely as a result of the fall in IL-23 levels.

The cytokine CCL2 is expressed on the intestinal epithelium of nematode-infected mice and is known to induce the attraction of mucosa-like mast cells^26^. The results for the *A. tetraptera*-infected control mice (C-IF) showed a stronger CCL2 fluorescence signal, both in the lamina propria as well as in the crypts. This high CCL2 fluorescence signal on the surface of the intestinal epithelium of infected mice is a consequence of the Th2 induction after a nematode infection.

Another regulator that stimulates the Th9 response is the cytokine OX40^52^, which is a CD134 ligand that plays a key role in the activation and proliferation of T lymphocytes serving as a chemoattractant. OX40 is a co-stimulatory molecule of the tumour-necrosis factor receptor superfamily (TNFR)^53^ that regulates CCL20 production^54^. In general, this cytokine provides a second stimulation pathway, helping to reinforce T-cell functions^55,56^. Stimulation with OX40 increases the expression of IL-21 and the receptor for IL-23 in Th17 cells. Accordingly, our results indicate that the mice injected with the IL-23^mAB^ registered the highest fluorescence levels corresponding to OX40, particularly the infected group (AB-IF), with the signal appearing both in the lamina propria as well as in the crypts. The greater expression of the chemokine OX40 in the mice treated with the IL-23^mAB^ could be due to a feedback mechanism in order to express more IL-23R as a means of palliating the functional IL-23 deficit in the mice neutralized with the IL-23^mAB^. However, recently, it was demonstrated that OX40 is a potent repressor of Th17 cells and the autoimmune diseases mediated by these cells. Under conditions of Th17 polarization *in vitro*, the reinforcement with OX40 inhibits IL-17 production despite greater survival and proliferation of the cells, and this inhibition could be due to an epigenetic regulation process that inhibits IL-17 production^57^.

The immunohistochemistry experiments examining the accumulation of IL-17 in the intestinal mucosa of the different groups of mice show similar areas of fluorescence for the infected groups (C-IF and AB-IF), which are larger than the areas of the uninfected control groups (C and AB-C), as shown in Fig. 4c. This expression of IL-17 could come directly from the CD11b^+^ dendritic cells^17^ stimulated by the antigens of the nematode and could be an independent action of the IL-23 on Th17. In this sense, Lee *et al*.^58^ demonstrated the protective effect that IL-17A exerts on the intestinal permeability after mucosal damage, an effect manifested regardless of the action of IL-23 on the Th17 cells^58^. Given that *A. tetraptera* is a parasite that does not cause appreciable damage at the intestinal level, its presence alone when infecting the mice would induce IL-17 in the mucosa regardless of the response of the Th17 cells.

The expression levels of IL-21 in the different groups of experimental animals differ depending on the tissue. While the spleen in the three experimental groups (C-IF, AB-IF and AB-C) has very low expression levels compared to the C group, the AB-C group registered the highest expression values of IL-21 in the MSN. In contrast, in the PP, the infected control (C-IF) and the groups in which IL-23 was neutralized, infected or not infected (AB-IF and AB-C), showed highly significant levels with respect to the control group (C). The high IL-21 production in the PP in the infected groups might be due to the direct action of the parasite on the intestinal mucosa. However, the mRNA-expression values for this interleukin among the IL-23^mAB^ groups of mice (AB-C and AB-IF) implies that it is not completely dependent on the stimulus caused by the parasitism, given that in the AB-IF group, IL-21 production was stimulated, although its values were not highly significant.

As occurs with IL-9, the pleiotropic action of IL-21 acts in a similar way as OX40, strengthening the expression of the IL-23 receptor (IL23R), which in turn bolsters the action of IL-23^59^. This would explain, by a regulatory mechanism, the high IL-21 expression in the PP in the groups of mice treated with IL-23^mAB^. However, it is especially activated by Th17 cells, inhibiting the phagocytic capacity of the dendritic cells (DC) and the antigen-presenting cells (APCs), blocking the capacity of mast cells to release inflammatory mediators, while inhibiting the proliferative ability of the Treg cells and activating the production of antibodies in B cells^60^. On the other hand, IL-21 acts directly on the intestinal epithelial cells to induce the expression of the chemokine CCL20 capable of attracting the cells that express the CCR6 receptor of the Th1 and Th17 cells^61^. These findings suggest that the parasitism by nematodes, even one as unaggressive as *A. tetraptera*, can induce a response at the intestinal level and enable the expression of CCL20, as well as IL-17 itself, or IL-6^62^. In fact, IL-6 is significantly expressed in the PP in the infected groups and especially in the C-IF group. Given that IL-21 induces the expression of the IL-23R, IL-21 might be responsible for the regulation and maintenance of the expression of the Th17 response and specifically of IL-17. Although the role of IL-21 may involve the immunoglobulins of the B cells, IL-21 can induce CD40 in the naïve B cells to induce the IgA isotopes, inhibited by IL-4^63^. IL-21 also plays a role in the activation and expansion of plasma cells^64^. CD138 is a receptor in the plasma cells, and the relationships between IL-23 and plasma cells remain poorly studied^65^. In our results, the CD138 levels were similar to those found for IL-21 interleukin, where the maximum fluorescence was observed in the infected control groups treated with IL-23^mAB^. Cocco *et al*.^65^ showed that plasma cells possess a receptor for IL-23 and significantly up-regulate IgM secretion when cells were treated with IL-23. On the other hand it has been determined that mast cells are directly responsible for the changes in intestinal permeability that occur in animals with high levels of IL-9, by action of mast cell enzymes on the occludin that cements the intestinal epithelium^18^.

In addition, IL-9 induces the expression of CCL20 in lung epithelial cells, where it mediates the recruitment of DCs tCD8^+^, Cytolytic T Lymphocytes (CTLs) cells through CCR6, which are expressed in these cells. CCL20 is involved both in the inflammatory response as well as in the homeostatic response that recruits B cells, which also express CCR6^66^. Recently, the expression of CCL20 was described to be associated with the stimulation of TLRs^67^, and it is likely that this increase in CCL20 is the consequence of TLR3 stimulation by the *Aspiculuris* antigens, similar to what occurs in the parasitism by other nematodes^68–70^. In fact, in the treatment of Crohn’s disease using embryonated *Trichuris suis* eggs, the release of antigens concomitant to the hatching and the onset of nematode development is enough to diminish the symptoms of the disease^65^. Recently, it was observed that the high levels of TGF-β downregulate CCL20^71^, whose levels are very high in ulcerative colitis and in Crohn’s disease^67^ and in our experiments in the PP of the AB-C group with minimum values of CCL20 in the mucosa.

As with OX40, the CCL17 cytokine is necessary for the induction of an inflammatory response in the mouse intestine, with the activation of Th1/Th17 reducing the expansion of the Treg cells^72^. However, CCL17 is the major chemoattractant for the Th2 T cells^73^.

In dendritic cells stimulated with IL-25, the CCL17 cytokine is rapidly induced, which in turn attracts T cells that produce IL-9. IL-25, a cytokine present in the mucosa, is produced by stressed epithelial tuft cells^74^. IL-25 rapidly activates the production of IL-5 and IL-13, the latter of which plays an important role in intestinal nematode and trematode expulsion^75–80^.

However, when the other attractant of neutrophils, GRO, was studied, it was found that the C-IF group showed significantly high fluorescence values, with a fluorescence distribution around the mucosa, while in the AB-IF group, the expression levels of the GRO were lower than in the AB-C group, suggesting that *A. tetraptera* induces an inflammatory process at the level of the mucosa that is diminished when IL-23 is neutralized.

In the intestinal epithelium, a recent study showed that sentinel tuft cells^71^ are capable of detecting the presence of bacteria or parasites, increasing their number in response to the infiltration of pathogens^10,81,82^, and thereby participating in the immune response against parasites. Tuft cells are currently considered the prime source of parasite-induced IL-25 production, and IL-25 promotes the production of IL-13 which induces as well IL-4, and the production of CCL17 that amplifies the Th2 response^75^. In our study, high levels of IL-25 were observed in the C-IF group in the PP and in the MSN and also CCL17 in the intestinal mucosa. Importantly, here we observe that pre-treatment of infected animals with anti-IL-23 completely reduces the rise in IL-25 levels observed during the infection in both PP and especially MSN.

The role of tuft cells has also been correlated with the presence of eosinophils^81^, an indirect way of correlating with intestinal parasitization and with the higher levels of the IL-5, because eosinophilia is associated with parasitism^83^ which increases peripheral blood in a Th2 environment^84^.

The relation between IL-17 and the Th2 immune response is complex. In this sense, animals deficient in IL-17 are incapable of expressing IL-13 when infected with the nematode *Nippostrongylus brasiliensis*^85^, implying that IL-17, either directly or indirectly, through the intervention of IL-25 or IL-9, promotes a Th2 response. In addition, treatment of T cells with IL-25 bolsters the expression of IL-9^13^, implying that the effect of expelling the worms attributed to the IL-25 would potentially be mediated by IL-9, capable of inducing changes in the intestine, as mentioned above^86^.

In summary, the low IL-23 levels determine the environment required for avoiding the implantation of the nematode larvae recently established in the mucosa of the intestine. This is perhaps a result of the stimulation of the binomial IL-25/IL-13 in an environment deficient in IL-17, due to the increase in Th2 – Th9 interleukins and the cascade of effects both at the physiological as well as the immunological levels that impede the implantation of the parasites.

Taken together, our results add support to the existence of a mechanism of Th2 induction in the absence of IL-17 provoked by the antibody-mediated depletion of IL-23, with a possible concomitant regulation of IL-17/Th2. Our present work describes for the first time that this mechanism operates in the intestinal mucosa. The changes in the levels of CCL2, CCL17, CXCL15, CCL20, OX40, tuft cells, CD138, and mucus observed upon treatment are in agreement with the elevation of the Th2 response, demonstrating the regulatory mechanism Th17/Th2 described, preventing the implantation of the intestinal nematode in mice after intraperitoneal administration of the anti-IL-23 antibody. In support of this hypothesis, the proposed mechanism is similar to the one recently described in lung dermis in asthma and in atopic dermatitis, where IL-17 induces a Th2 response^87–89^. However, in all cases, the exact mechanism underlying this regulation still needs to be determined.

In conclusion, in addition to the insight provided regarding the interplay between cytokines described above, our data indicate that the ablation of IL-23 may be a powerful approach for the control of helminth diseases and although further work with different parasites in other experimental models is required, the work presented here offers an exciting framework for future studies.

## Materials and Methods

### Mice and parasites

Male mice of the inbred CD1/ICR strain, 4–6 weeks of age, were housed under specific pathogen-free conditions in animal facilities with *ad libitum* access to food and water, regulated temperature, and controlled light/dark cycle conditions. All mice were weighed weekly throughout the course of the experiments. This study was performed in strict accordance with the recommendations of the Ethics Committee of Experimental and Animal Welfare of the Universitat de València, which approved the protocol (2015/VSC/PEA/00069 type 2).

For infection, *A. tetraptera* gravid females were isolated from naturally infected mice. The mice were sacrificed by cervical dislocation and their large intestines were removed and longitudinally opened. The *A. tetraptera* females were washed and kept at 24 °C in PBS containing 100 U penicillin and 100 μg/mL streptomycin (Sigma-Aldrich), for seven days, in order to obtain an adequate amount of intrauterine embryonated eggs^78^. After the embryonated eggs were counted under a stereoscopic microscope, 100 eggs were orally administered to each mouse of the various experimentally infected groups.

### Experimental design

A total of 20 male mice were divided into four groups of 5 animals each: (i) Uninfected and untreated control group (C); (ii) control infection group, (C-IF group), mice experimentally infected; (iii) antibody injected-infected group (AB-IF group), in which mice were injected intraperitoneally with 6.6 µg of anti-IL-23 monoclonal antibody (IL-23^mAB^) (anti-mouse IL-23 p19 purified, eBioscience, No 14-7232) one and two days prior to infection and on 6 consecutive days after infection; iv) and the AB-C group, injected intraperitoneally with 6.6 µg of the IL-23^mAB^ as described before, but uninfected.

Mice were sacrificed by cervical dislocation 23 days after the challenge infection. The colon of each mouse was examined and its parasite load was determined. Spleen, mesenteric lymph nodes (MSN), and Peyer’s patches (PP) were excised, and placed in RNAlater^®^ (Qiagen-76104) buffer (1:5), an RNA-stabilization reagent, for further cytokine measurement and stored at −80 °C. Additionally, a section of the proximal colon from each mouse was removed and placed in a solution of 0.5% glutaraldehyde and 2.5% paraformaldehyde in PBS, pH 7.4, for further immunohistochemical analysis.

### RNA isolation, real-time PCR for quantification of cytokine mRNAs

Total RNA from 20–30 mg of spleen, MSN or PP were purified using the RNAeasy Midi kit (Qiagen-74106) following the manufacturer’s instructions and each sample was digested with RNase-Free DNase Set (Qiagen-79254) in order to remove DNA contamination. The Oligotex^®^ mRNA Midi kit (Qiagen-70042) was used subsequently for mRNA isolation. The quantity of total RNA and the purified mRNA was determined using a Nanodrop ND-1000 (Thermo Scientific), and the quality was determined using an Experion automated electrophoresis system (Bio-Rad, Nazareth Eke, Belgium). Then, between 100 fg and 1 µg of mRNA from each sample, were reverse transcribed into cDNA using a mix of oligo-dT and random primers to amplify all the mRNA in a CFX96 real-time system (Bio-Rad) with the iScript cDNA Synthesis kit (Bio-Rad, 170–8891), as previously described^70^. The concentration and quality of the cDNA was calculated spectrophotometrically in a Nanodrop (ND-1000, Thermo Scientific). The cDNA was diluted 1:10 and stored at −80 °C.

The cytokine expression (IL-2, IFN-γ, IL-12 p35, IL-15, IL-6, TNF-α, IL-4, IL-10, IL-13, TGF-β, IL-17, IL-23 p19, IL-25, IL-21, and IL-9) was analysed by quantitative real-time PCR (qPCR). For this, 50 ng of each cDNA were amplified using primer pairs and probes specific for the respective cytokine, which were specifically designed using the eprimer3 software, as described in Table S1^70^. The primer concentration was optimised and dissociation curves were generated for each pair of primers to verify the amplification of a single PCR product. The thermocycler CFX96 Real-Time System (Bio-Rad) and PCR System (Applied Biosystems) were used to perform the amplification reactions in 96-well plates, consisting of an initial setup of 2 min at 95 °C, followed by 40 cycles of 10 s for denaturation at 95 °C, 30 sec of annealing at 55 °C and 1 min of extension at 60 °C. Samples were kept at 12 °C after the amplification. In each plate, samples, endogenous control, and negative control were analysed in triplicate. Expression of β-actin was used to normalize gene expression (∆∆C(t)) analysis. Expression levels of each cytokine of the treated animals were relativized with respect to the untreated control group (group C).

### Immunohistochemistry of mouse intestine

For histological evaluation, the glutaraldehyde/paraformadehyde-fixed piece of the first section of the colon was processed. The samples were embedded in paraffin and sections of 5–10 µm were cut and attached to slides. Then, the paraffin was removed by three dips of 15 min in xylene. For hydration, the samples were dipped in a decreasing gradient of ethanol (100%, 90%, and 70%) and in water for 15 min at room temperature. The slides were then placed in 0.01 M citric acid at pH 6.0 and heated at 120 °C for 10 min in a microwave for antigen retrieval. To prevent the non-specific binding of the antibodies, the slides were blocked for 30 min with PBS containing 1% of albumin from chicken egg white (Sigma, A5503) and then treated with different specific antibodies at a dilution of 1:50 in blocking solution for 1 h at room temperature. To detect oligosaccharides containing terminal N-acetylglucosamine in glycoproteins and mucus-rich areas of the crypts in the colon mucosa, WGA lectin labelled with fluorescein was used. Antibodies recognising chemokines CCL2, CCL17, CXCL15, CCL20, OX40, Growth-Regulated Oncogene-alpha (GRO), and IL-17 were also used. In addition, the plasma-cell (anti-CD138) and tuft cell (DCAMKL-1antibody) markers were also employed. When the primary antibodies were not labelled, specifically labelled secondary antibodies were used as indicated in Table S2.

Additionally, cell nuclei were stained for 10 min in DAPI solution (10 μg/ml) (4′,6-diamidino-2-phenylindole dihydrochloride) (Sigma, D9542). The slides were subsequently stored and mounted in a mounting medium (Prolong Antifade Lit, Molecular Probes) and examined under a Leica DMI6000 confocal laser microscope equipped with a filter system for FITC. Images were analysed with the ImageJ program. The fluorescence intensity was measured four times in an area of 100 µm^2^ and the number of fluorescent cells was quantified.

### Statistical analysis

To determine the statistical significance of the results, the Tukey-Kramer multi-comparisons test was used. Results are expressed as the mean ± SEM and differences in mean values were considered statistically significant when *P* < 0.05. For statistical analysis, Graph-PAD INSTAT v.3.05 software (Graph Pad Software, Inc., La Jolla, CA, USA) was used.

## Electronic supplementary material

Supplementary information
