# Supplementary material for: Intraperitoneal administration of the anti-IL-23 antibody prevents the establishment of intestinal nematodes in mice
Source: Sci Rep. 2018 May 17;8:7787. doi: 10.1038/s41598-018-26194-x (PMC5958071; doi:10.1038/s41598-018-26194-x)
Supplement: Supplementary file 1 — Supplementary information [file 41598_2018_26194_MOESM1_ESM.docx]

**Intraperitoneal administration of the anti-IL-23 antibody prevents the establishment of intestinal nematodes in mice**

M. Gomez-Samblas^1^, D. Bernal^2,^ A. Bolado-Ortiz^3^, S. Vilchez^4^, F. Bolas^5^, A.M. Espino^6^, M. Trelis^3^, A. Osuna^1*^

Table S1. Primers and probes sequence of cytokines genes analyzed

| Primer | Sequence (5'- 3') |  |
| --- | --- | --- |
| β-Actin | Sense | TCCATCATGAAGTGTGACGT |
|  | Antisense | GAGCAATGATCTTGATCTTCAT |
| IL-2 | Sense | CCACTTCAAGCTCCACTTCA |
|  | Antisense | ATCCTGGGGAGTTTCAGGTT |
| INF-γ | Sense | GCTCTTCCTCATGGCTGTTT |
|  | Antisense | GTCACCATCCTTTTGCCAGT |
| IL-12 | Sense | GACCAAACCAGCACATTGAA |
|  | Antisense | CTACCAAGGCACAGGGTCAT |
| IL-15 | Sense | CATTTTGGGCTGTGTCAGTG |
|  | Antisense | TGCAACTGGGATGAAAGTCA |
| IL-6 | Sense | AGTTGCCTTCTTGGGACTGA |
|  | Antisense | TCCACGATTTCCCAGAGAAC |
| TNF-α | Sense | CCCCAAAGGGATGAGAAGTT |
|  | Antisense | CACTTGGTGGTTTGCTACGA |
| IL-4 | Sense | CCTCACAGCAACGAAGAACA |
|  | Antisense | ATCGAAAAGCCCGAAAGAGT |
| IL-10 | Sense | CAGAGCCACATGCTCCTAGA |
|  | Antisense | TCATTTCCGATAAGGCTTGG |
| IL-13 | Sense | AGCATGGTATGGAGTGTGGA |
|  | Antisense | TTGCAATTGGAGATGTTGGT |
| TGF-β | Sense | TGGAGCAACATGTGGAACTC |
|  | Antisense | AGCCCTGTATTCCGTCTCCT |
| IL-17 | Sense | TCCAGAAGGCCCTCAGACTA |
|  | Antisense | TCATGTGGTGGTCCAGCTT |
| IL-23 | Sense | TAATGTGCCCCGTATCCAGT |
|  | Antisense | AGGCTCCCCTTTGAAGATGT |
| IL-25 | Sense | CGGAGGAGTGGCTGAAGTGGAG |
|  | Antisense | ATGGGTACCTTCCTCGCCATG |
| IL-21 | Sense | GAGGACCCTTGTCTGTCTGG |
|  | Antisense | TCATCTTTTGAAGAAGCCATTT |
| IL-9 | Sense | TGATTGTACCACACCGTGCT |
|  | Antisense | AGGTCACTCCAACGATACGG |

Table S2. Antibodies and lectin used in immunohistochemistry.

| **Primary antibody** | Fluorochrome | Cat. Nº | **Secondary antibody** | Fluorochrome | Cat. Nº |
| --- | --- | --- | --- | --- | --- |
| anti-CD134 / OX40 [OX-86] | FITC green | Ab33998 |  |  |  |
| anti-CCL20/MIP3 alpha |  | Bs1268R | Goat anti-Rabbit IgG (H+L) | Alexa Fluor^®^ 647 | A-21247 |
| anti-mCCL2/JE/MCP-1 |  | AF479-NA | Donkey anti-goat (H+L) | Alexa Fluor^®^ 594 | AF-150132 |
| anti-mCCL17/TARC |  | AF529 | Donkey anti-goat (H+L) | Alexa Fluor^®^ 594 | AF-150132 |
| anti-CXCL15/Lungkine |  | AF442 | Goat anti-Rabbit | FITC | F-0382 |
| anti-GRO/ CXCL1 |  | Ab17882 | Goat anti-Rabbit | FITC | F-0382 |
| anti-Dcamkl1CAMKL1 antibody |  | Ab31704 | Goat anti-Rabbit IgG (H+L) | Alexa Fluor^®^ 647 | A21245 |
| anti-mouse CD138 |  | 142502 | Goat anti-Rat IgG | FITC | F-6258 |
| anti-IL-17 |  | Ab79056 | Goat anti-Rabbit | FITC | F-0382 |
| **Lectin** |  |  |  |  |  |
| Lectin from *Triticum vulgaris* (WGA) | FITC | L4895 |  |  |  |
